# New journal authorship criteria: how *e*cancermedicalscience is supporting authors and readers from underserved settings

**DOI:** 10.3332/ecancer.2020.ed106

**Published:** 2020-09-18

**Authors:** Eduardo Cazap, Richard Sullivan, Katie Foxall

**Affiliations:** 1 Editor-in-Chief, ecancer, 13 King Square Avenue, Bristol, BS2 8HU, UK; 2 President, Latin American and Caribbean Society of Medical Oncology (SLACOM), Buenos Aires, Argentina; 3 *e*cancer Chair of Trustees; 4 Institute of Cancer Policy, Kings Health Partners Comprehensive Cancer Centre, King's College London, London, UK; 5 Head of Publishing, ecancer, 13 King Square Avenue, Bristol, BS2 8HU, UK

**Keywords:** open access publishing, LMICs, global health and cancer

## Abstract

In order to reduce the increasing cancer burden in Lower and Middle Income Countries (LMICs), oncology journals must support authors from underserved settings to become fully involved in the global publication system, without facing barriers to publishing their research such as geographical bias and lack of funding. ecancermedicalscience’s goal has always been to publish high-quality research which contributes towards narrowing the gap between those who have access to adequate cancer prevention, treatment and care and those who do not. The time is now right for the journal to take new steps in proactively supporting authors from LMICs and the global partnerships that are vital to increasing the availability of resource-appropriate data. With this in mind, ecancermedicalscience will only be accepting submissions which feature at least one author from an LMIC, or which have a significant impact on under-resourced settings.

The World Health Organisation (WHO) has estimated that 81% of new cancer cases over the next two decades will occur in LMICs, where survival rates are currently lowest, but only 5% of total global spending on cancer care is expended in these regions [1]. There is a general lack of awareness among policy makers and the general public concerning the magnitude of the current and future cancer burden and its impact.

This highlights the urgent need to increase the amount of education regarding cancer prevention and treatment in underserved settings, and provide policy makers with high-quality, peer-reviewed, regionally appropriate evidence. Increasing the amount of research which is published and read by oncology professionals in LMICs is key to raising awareness of the latest developments in global cancer research and bringing attention to the region-specific issues surrounding cancer prevention and care in underserved settings. The dissemination of innovative and practical global cancer information is a fundamental component of reporting practices which adapt existing health infrastructure to resource-constrained settings. Even in High Income Countries, there are underserved populations such as people living in poverty, migrants and indigenous people who are at higher risk of cancer and have poorer outcomes – more research needs to be carried out and published about these groups in order to reduce the existing health disparities.

The World Cancer Declaration calls for the provision of innovative education and training for healthcare professionals (HCPs) to improve significantly, particularly in LMICs [2]. The inequality of global publication systems represents a serious obstacle - research from the global North is seen as ‘mainstream’ or ‘international’ and research from the developing world is of ‘local’ interest only and therefore lacking sufficient impact for inclusion in the system unless it addresses issues that are of specific interest to Northern readers [3]. In 2017, five times as many research publications were published by the top ten countries in the world than by the bottom 200 [4]. As an example, Africa makes up around 15% of the global population, but only 0.7-1% of the world’s published research is by African authors [5].

Researchers from Africa and other LMICs are the least able to pay to access information or afford open access Article Processing Charges (APCs) so that they can publish their own research, resulting in ‘lost science’ in the form of information which is either not published or simply not made freely accessible to all. This leads to doctors being unaware of specific data and best practices which would make a difference to outcomes for their patients.

The results of a survey of African oncology HCPs which *e*cancer carried out in 2019 showed that the majority of the 85 respondents (78%), had faced barriers to getting their research published, generally due to a lack of funding, inadequate research techniques, geographical bias and linguistic difficulties. A lack of available data which was relevant to their regional or racial context was also a problem. 85% had faced problems accessing the latest research and guidelines in order to be able to treat their patients—the biggest obstacle was cost [6].

More recently, a new challenge faces cancer control improvement worldwide. The explosion of the COVID-19 crisis has changed the picture: the urgency of controlling the pandemic has delayed or complicated cancer prevention and care, particularly in under-resourced settings. In this new context, rapid global information is a critical need. The inclusion of an innovative fast-track process for cancer and COVID-19 papers, with an average review process of 14 days, demonstrates the flexibility and rapid innovation of *e*cancer’s Editorial team.

We have taken the decision to refine *e*cancermedicalscience’s submission criteria to only accept articles with at least one author based in an LMIC or representing research that directly benefits LMICs. This pioneering approach will allow us to contribute towards addressing the imbalance in global scientific publishing and combat the significant lack of data on which to base national cancer control plans in LMICs. We feel that the time is right to proactively support authors from LMICs and the global collaborations that are vital to the development of key skills in this area. Our hope is that this will encourage fruitful partnerships between High Income Countries and LMICs to progress the pace of cancer research globally. The journal has always been fully open access and this will continue, so that no reader is barred from accessing the information they need.

We will focus on commissioning articles which report new developments and show best practice in subjects which most urgently need to be covered for underserved settings, such as the implementation of vaccinations for liver and cervical cancers, tobacco-control policies for smoking-related cancers, and low-tech early detection methods for cervical cancer, as well as pain relief at the palliative stage.

It is essential that local experts are involved at every step of the publication process, so selected experts in these areas are invited to join the Editorial Board and asked to serve as Guest Editors on special issues focusing on under-resourced settings. The journal team works closely with authors during the pre-submission, peer review and publication processes, offering personalised support and helping them to develop their papers to meet international publishing standards. The publishing system has always been biased towards those authors who have the benefit of training and mentorship from colleagues and their institutions so at *e*cancer we help to close this gap by offering the extra support some authors from LMICs need to develop a high-quality manuscript. We also provide free English language editing where necessary, and authors are able to submit in Spanish—if their article passes peer review it will be translated for free and published in both English and Spanish. Only peer reviewers with a deep understanding of underserved settings are selected for these articles. All authors also receive free copyediting and open access publication with a CC BY copyright licence.

## Conclusion

The international publishing community has a duty to support and collaborate with researchers and clinicians in underserved settings, with a consequent positive impact on population health and patient outcomes. The new submission criteria for *e*cancermedicalscience will focus our resources on making a significant contribution to ramping up scholarly output to help support the healthcare communities who are on the front line of the current and future crisis in cancer care and treatment. Every patient deserves the best possible care and at *e*cancermedicalscience we will continue to foster international communication and collaboration and offer the support that will empower healthcare communities in the worst affected areas.

## Conflicts of interest

KF is employed by *e*cancer, UK charity no. 1176307. There are no other conflicts of interest to declare.

## Funding declaration

No direct funding for this article was received.
